# Anomaly Detection in Railway Sensor Data Environments: State-of-the-Art Methods and Empirical Performance Evaluation

**DOI:** 10.3390/s24082633

**Published:** 2024-04-20

**Authors:** Michał Bałdyga, Kacper Barański, Jakub Belter, Mateusz Kalinowski, Paweł Weichbroth

**Affiliations:** 1 Meritus Systemy Informatyczne Sp. z.o.o., Prosta 70, 00-838 Warsaw, Poland; 2 Department of Software Engineering, Faculty of Electronics, Telecomunications and Informatics, Gdansk University of Technology, 80-233 Gdansk, Poland

**Keywords:** anomaly detection, systematic literature review, sensor data environment

## Abstract

To date, significant progress has been made in the field of railway anomaly detection using technologies such as real-time data analytics, the Internet of Things, and machine learning. As technology continues to evolve, the ability to detect and respond to anomalies in railway systems is once again in the spotlight. However, railway anomaly detection faces challenges related to the vast infrastructure, dynamic conditions, aging infrastructure, and adverse environmental conditions on the one hand, and the scale, complexity, and critical safety implications of railway systems on the other. Our study is underpinned by the three objectives. Specifically, we aim to identify time series anomaly detection methods applied to railway sensor device data, recognize the advantages and disadvantages of these methods, and evaluate their effectiveness. To address the research objectives, the first part of the study involved a systematic literature review and a series of controlled experiments. In the case of the former, we adopted well-established guidelines to structure and visualize the review. In the second part, we investigated the effectiveness of selected machine learning methods. To evaluate the predictive performance of each method, a five-fold cross-validation approach was applied to ensure the highest accuracy and generality. Based on the calculated accuracy, the results show that the top three methods are CatBoost (96%), Random Forest (91%), and XGBoost (90%), whereas the lowest accuracy is observed for One-Class Support Vector Machines (48%), Local Outlier Factor (53%), and Isolation Forest (55%). As the industry moves toward a zero-defect paradigm on a global scale, ongoing research efforts are focused on improving existing methods and developing new ones that contribute to the safety and quality of rail transportation. In this sense, there are at least four avenues for future research worth considering: testing richer data sets, hyperparameter optimization, and implementing other methods not included in the current study.

## 1. Introduction

The first steam locomotive undoubtedly heralded a transformative era, and since their inception in the early 19th century, railways have remained central to public transport [1]. Recently, the potential of railways to alleviate road and air congestion and environmental challenges has brought them back into the spotlight [2]. In particular, there has been a noticeable increase in rail traffic across Europe for both passenger and freight transport [3,4]. Between 1990 and 2007, passenger kilometers increased by 28%, while freight ton kilometers increased by 15% in the EU-15 countries [5]. Worldwide, rail networks carried more than 3.5 trillion passenger kilometers in 2019, with China, India, and Japan leading in passenger traffic [6]. Meanwhile, European railways recorded around 643 billion passenger kilometers in the same year [7]. On the economic front, the global rail freight market was valued at $247.4 billion in 2020, with projections of growth to nearly $280 billion by 2026 [8].

In the railway sector, the integrity of train wheels is of paramount importance. Various defects such as wheel flats, spalling, chipping, and polygonization are common [9,10]. Advances in sensor technology, driven by the integration of the Internet of Things (IoT) and Artificial Intelligence (AI), have revolutionized monitoring and diagnostics in various industries, including construction [11], energy [12], healthcare [13], renewable energy [14], security [15], and transport [16]. Specifically in the railway context, a typical train bogie can house between 10 and 50 sensors. Among these, acoustic sensors are critical as they monitor vibrations and help in the early detection of anomalies in railway components. Such anomalies, if left unchecked, could lead to catastrophic consequences such as derailment.

Traditional monitoring approaches often struggle with the complex patterns of anomalies manifested in the time-series data generated by these sensors. However, the introduction of deep learning techniques to railway monitoring has yielded promising results, catalyzing the development of models capable of processing and interpreting vast amounts of data [17], in particular identifying unusual or unexpected events.

The plethora of anomaly detection methods still raises the need not only for their qualitative review and evaluation but also for their empirical performance evaluation. As a result, researchers are compelled to engage in comprehensive analyses that not only examine their design and current applications but, more importantly, delve into empirical evaluations of their performance. This growing demand underscores the critical importance of refining and improving anomaly detection techniques to ensure their effectiveness when applied to real-world datasets. Furthermore, the dynamic nature of data landscapes requires a continuous and adaptive approach to the evaluation of anomaly detection methods, fostering continuous evolution in the field. However, to the best of our knowledge, there are few studies that have undertaken similar efforts. Thus, our study attempts to fill this gap by conducting a systematic literature review followed by experimental research.

The rest of the paper is structured as follows. In Section 3, we elaborate on the details regarding the design the qualitative research, followed by the discussion on the results obtained in Section 4. Next, in Section 5, we present the results obtained from performed performance evaluation. In Section 2, we analyze the related work, and then we present a discussion in Section 6. Eventually, in Section 7, we conclude the paper.

## 2. Related Work

This section delves into the research articles that have successfully navigated our comprehensive search strategy. The discussion is segmented into five distinct categories, identified during our rigorous review process. These primary categories encompass the methodologies proposed in the literature: Deep Learning Techniques, Time-Series Analysis Methods, Wavelet-Based Approaches, Statistical Inference Models, and Miscellaneous Techniques. The last category aggregates pivotal works that, while not fitting neatly into the aforementioned classifications, provide invaluable insights pertinent to the domain of railway anomaly detection using vibration sensor data.

In the realm of railway infrastructure monitoring, the role of sensors and their integration with advanced anomaly detection methods is paramount. Origlia et al. [17] utilized accelerometric sensors on train vehicles to detect anomalies. Their approach involved a comparative analysis of three self-supervised models, emphasizing both predictive and reconstructive approaches to gain insights into time series data.

Xu et al. [18] introduced unsupervised causality-based feature extraction methods for fault detection in high-speed trains. Their methods aimed to extract useful information from high-dimensional monitoring data collected from sensors, adopting a score-based method for causal discovery using the BIC score and hill-climbing algorithm. This approach contrasts with Park et al. [19], who proposed a health index for the early detection of ball bearing faults using a microphone sensor. Their method introduced the Frequency Energy Shift Method (FESM) to extract a health index suitable for detecting incipient faults and tracing its progression over time.

Islam et al. [20] presented a novel anomaly detection system for the Internet of Railways (IoR) using extended neural networks. Their methodology employed k-means clustering for feature scoring and ranking in the dataset, with the aim of enhancing classifier performance in an unsupervised manner. Zuo et al. [21] combined classical time-domain features with scale-averaged wavelet power (SAWP) to process vibration data. Their signal-processing procedure involved extracting features from both the time domain vibration signal and the SAWP, using the isolation forest algorithm for squat detection in railway switches and crossings.

Steenwinckel et al. [22] introduced the FLAGS methodology, which integrates expert knowledge with machine learning for adaptive anomaly detection on sensor data streams. Their approach utilized the Matrix profile technique as a machine learning anomaly detection module to detect outliers in the raw data. Meanwhile, Hesser et al. [23] focused on monitoring and tracking a suspension railway using data-driven methods applied to inertial measurements. Their methodology utilized computational intelligence to recognize features of the characteristic track profile, employing a k-means clustering algorithm for data labeling and an Artificial Neural Network (ANN) for precise localization.

Vos at al. [24] emphasized the significance of handling imbalanced datasets in anomaly detection, particularly when anomalies are rare. They introduced a deep learning method combining LSTM architectures with a one-class SVM to distinguish abnormal data from normal vibration signals. Their methodology involved using computational intelligence to process raw data, with the one-class SVM model trained on healthy-only data. This approach was applied to vibration signals collected during endurance tests of gearboxes and aircraft test flights.

Cunha et al. [25] presented a comprehensive review of machine learning (ML) methods applied to structural dynamics and vibroacoustic (SD&V). The paper emphasized the significance of ML in modeling physical phenomena in SD&V, especially when traditional models are either unknown or computationally challenging. They highlighted the role of ML in Structural Health Monitoring (SHM), the active control of noise and vibration, and vibroacoustic product design. The paper provided insights into the strengths and limitations of various ML methodologies in the context of SD&V, discussing the role of digital twins and physics-guided ML. The article also gave an overview of various ML methods, including neural networks, support vector machines, and Gaussian process regressors, and discussed the challenges faced in implementing ML in SD&V, such as the need for large labeled datasets and the computational cost of ML simulations in real-time applications.

Wan et al. [10] highlighted the importance of anomaly detection for train wheels, introducing an unsupervised data-driven workflow that uses the Short-Time Fourier Transform (STFT) to extract time-frequency features from vibration data. They employed a pair of fiber Bragg grating (FBG) sensors to collect this data. Their approach utilized four unsupervised learning algorithms, including OC-SVM and CNN-AE, to derive health indexes for monitoring train wheel conditions.

In conclusion, the collective studies underscore the growing importance and potential of using advanced data-driven methods and sensors for anomaly detection in railway systems. They highlight the innovative solutions proposed to address the challenges faced in the domain, setting the stage for further research and advancements.

## 3. Systematic Literature Review

This section describes the strategy adopted to identify relevant sources for the literature review. To this end, selected keywords, the search string, and the inclusion and exclusion criteria and the quality criteria used in the literature review are discussed.

### 3.1. Research Methodology

A systematic literature review (SLR) is a disciplined way of identifying, analyzing, discussing, and presenting the results of studies on a particular topic. More specifically, the purpose of carrying out an SLR is to provide an unbiased answer(s) for the research question(s) using an approach that is reliable, accurate, and verifiable. Accordingly, we adopted and adapted the guidelines elaborated on by Kitchenham [26,27,28].

### 3.2. Research Context and Goal

Since time series analysis, and in particular outlier (anomaly) detection, covers a wide range of domains, the context of our study lies in its current applications to the data environments of sensor devices. Here, a sensor device is a device that detects a physical quantity and responds by transmitting a signal. On this basis, the aim of this literature review is twofold: (a) to identify anomaly detection methods in time series applied to sensor device data, and (b) to recognise both the advantages and disadvantages of these methods.

### 3.3. Research Questions

In light of the above research context and objective, our review was guided by the following two research questions:

RQ1:  What anomaly detection methods have been applied to sensor data environments?
RQ2:  What advantages and disadvantages do these methods have?

These two research questions were used as input to identify and formulate the keywords necessary to design and implement a search strategy.

### 3.4. Keywords

Considering both research questions, a preliminary search revealed that only the first should have been considered to extract a set of keywords as the scope of the second does not typically cover the title, abstract, or author keywords in the research papers. We therefore considered only two keywords, namely, *anomaly* and *detection*. Furthermore, the aforementioned context was also taken into account as it was deliberately defined with the aim of limiting and positioning the scope of the review. To this end, the other extracted keyword was *sensor*.

### 3.5. Source Database

To achieve the aim of this study, we used the Scopus database in our review as it is one of the largest sources of searchable citations and abstracts of literature [29]. Due to its broad coverage of academic literature, we found it to be the most reliable, relevant, and up-to-date research data.

### 3.6. Inclusion Criteria

The following criteria for inclusion were adopted for the current study:

- The selection process strictly follows the search string (IC1).
- Only full-length articles are considered (IC2).
- The subject subject area covers computer science (IC3).
- The research is published in the English language (IC4).
- The time period covers the last five years that is 2018–2023 (IC5).

The above inclusion criteria were defined as the key features of the target population that we used to answer the research questions.

### 3.7. Exclusion Criteria

Different in nature to the inclusion criteria, the exclusion criteria concern characteristics that make the recruited population ineligible for the study. To this end, studies were excluded based on the below-defined criteria:

- Articles other than English are excluded (EC1).
- The availability of the document is restricted (EC2).
- The study did not concern any anomaly detection methods (EC3).

### 3.8. Search Execution

The entire search was carried out using the official Scopus website (http://scopus.com (accessed on 28 July 2023)). In the first run, we used the search string of the term *anomaly detection* in the category “Article title, Abstract, Keywords”. The server database found 34,588 documents. In the second run, the inclusion criteria were applied, which limited the results to 8318 articles. In the third run, the time frame was limited to the last five years (2018–2023) in order to focus on recent developments and trends rather than historical ones. This reduced the number of articles to 6274. In the fourth run, we added the third keyword (*sensor*) to the current string, indicating the logical AND relationship between them. In the end, 846 articles were submitted by the authors for quality assessment, which involved an in-depth content analysis with the aim of checking EC3. The final output included 461 papers.

**Figure 1:**
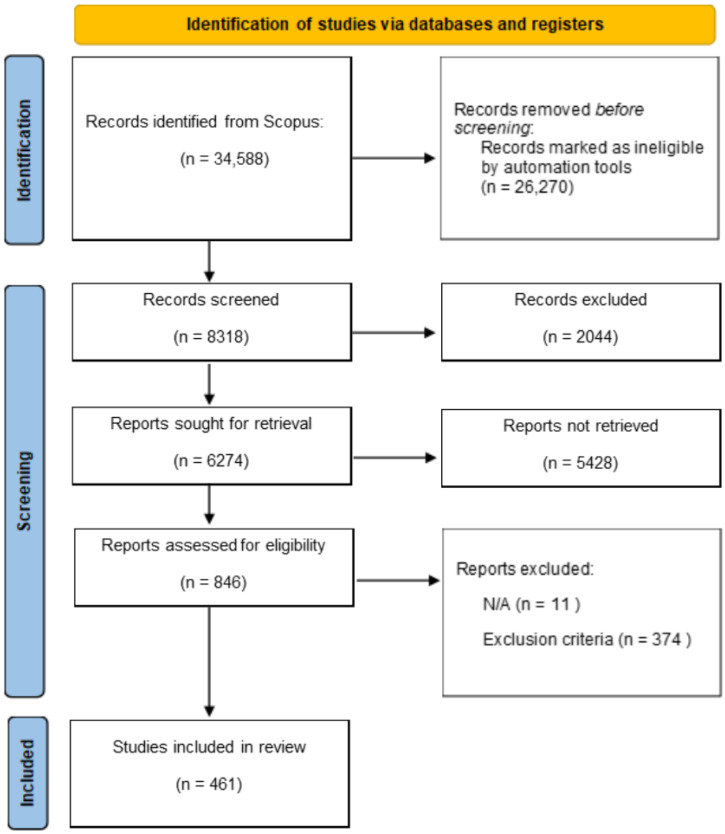
PRISMA flow diagram showing the process of selecting articles for review.

In summary, Figure 1 shows the review stages, and the corresponding results from each run, by using the PRISMA flow diagram [30].

### 3.9. Data Analysis and Synthesis

In this stage, two researchers separately analyzed the title, keywords, and abstract, in that order, to check whether any of the anomaly detection methods had been introduced. If one author found a method, the article was marked as positive, otherwise as negative. If both agreed, the article was classified for the next stage; if they disagreed, the third (senior) researcher was involved to discuss discrepancies and reach a consensus. In addition, if more than one method was identified, the full content of the article was examined.

In the case of the second research question, after reading each paper we looked back through the references to gain a better understanding of how knowledge on a topic has developed and to identify the topic experts. Afterwards, we examined the citations of each paper after its publication and screened the titles and abstracts. Therefore, both reverse snowballing [31] and forward snowballing [32] were performed. During this stage, an in-depth analysis process was conducted, which means that to a certain extent, all researchers were involved since the process was found to be a time-consuming and tedious effort. In addition, in order to capture the larger body of knowledge, two inclusion criteria (IC2 and IC5) were relaxed.

Due to the size and abundance of the knowledge extracted, we address the research questions in the next section.

## 4. Anomaly Detection Methods in Sensor Data Environments

### 4.1. Identified Methods

The preliminary check showed that there were numerous methods used with the aim of detecting anomalies. Therefore, in view of this relatively large output, we decided to adopt the classification of [33] since the generic nature of the types formulated at the first level of classification allows us to divide the sample and group items in a valid scheme. Similarly, our classification includes five types (see Table 1 for details). In the following, we will briefly discuss each of them.

The first type are statisticalmethods, which involve collecting, organizing, and analyzing data according to established principles to identify patterns, trends, and anomalies using descriptive statistics, inferential statistical analysis, and predictive analysis. In general, statistical approaches are driven by a data distribution model, and objects are evaluated in terms of how well they fit the model. The typical estimate of the distribution is the mean, but it is very sensitive to outlying values among the observations. A better choice is the median, which is more robust to an outlier values. There are 14 methods in this group.

The second type are clustering methods. The main advantage of clustering methods is their ability to learn from the data and recognize anomalies without explicit descriptions, specified by an additional attribute (a label), usually provided by an expert. By design, in other words, these unsupervised machine learning algorithms simply group the unlabelled data or data points into different clusters so that similar data points fall into the same cluster as those that are relatively different from the others. This group includes 10 methods.

The third type are classification methods. By its very nature, classification is essentially the process of understanding and predicting the class of new (uncategorized) observations on the basis of the training data. This approach is commonly known as supervised learning. Recently, several new approaches to anomaly detection have exploited classification through machine learning (deep learning) frameworks, which have achieved superior results. Another promising approach is ensemble learning, which improves the accuracy and robustness of prediction by combining the results of different models. In general, there are four subtypes within this group: the Support Vector Machine (SVM), Neural Networks, Ensemble Learning, and Others. In total, this group contains 29 methods and is the largest of all the others.

The fourth type concerns methods based on Information Theory. According to Shannon, a creator of Information Theory, a basic idea in Information Theory is that information can be treated very much like a physical quantity, such as mass or energy. It is a mathematical representation of the conditions and parameters that affect the transmission and processing of information [34]. Considering the problem of anomaly detection, the key idea is to take advantage of this theory to analyze time series provided by one or more sensors, which does not require prior knowledge of the system model. This group contains two methods and is the smallest of all the others.

The fifth and last type are hybrid and other methods. While the former are a combination of existing techniques, the latter include all other existing, adopted for anomaly detection. These methods represent complex computational models with the ability to be highly adaptive, distributed, and self-learning in nature. In total, this group contains 16 methods.

To sum up: a total of 71 methods were identified and classified into five different groups. These methods range from basic statistical methods, supervised and unsupervised machine learning to complex optimization systems. As different methods have been used over the years, it can be concluded that there is no single approach to detecting anomalies in sensor data environments.

### 4.2. Advantages and Disadvantages

With 71 methods identified as input, we discuss below the most commonly used methods to date, including 19 methods, considering the frequency of their use in anomaly detection.

#### 4.2.1. One-Class Support Vector Machine (OCSVM)

The OCSVM method is known for its proficiency in situations with scarce data, demonstrating versatility, maximizing the margin of separation, and exploiting hidden aspects of the data to improve generalization [35]. It uses dual-space projections, allowing for a more refined representation of the data. However, it is not without its drawbacks; the integration of additional detail can increase complexity, and there is an inherent limit to how deep one can go into a class using SVM+. Proper execution also requires meaningful data grouping, and managing group-related information remains a challenge.

#### 4.2.2. Local Outlier Factor (LOF)

The LOF algorithm stands out for its ability to efficiently detect anomalies in various data sets, underlined by its flexible applicability and its unique density-based approach [36]. This method provides insight into local density variations, making it particularly adept at distinguishing outliers in clustered data. However, LOF has its challenges. It can be memory intensive, not particularly agile when faced with changes, and struggles when applied to streaming data. In addition, its computational complexity can be a barrier in large-scale or real-time applications.

#### 4.2.3. Isolation Forest (IF)

Isolation Forest is a prominent algorithm, particularly favored for its efficiency in dealing with large datasets [37]. Not only is it adept at handling categorical data, but it also excels at finding anomalies, providing fast execution time, and effectively classifying outliers. Despite these strengths, it is not without its limitations. Compared to its *k*–means based counterpart, IF can be less accurate. In addition, it can sometimes struggle to detect inconspicuous points, and while its execution time is usually an advantage, there are instances where it becomes a disadvantage.

#### 4.2.4. Gaussian Hidden Markov Model (Gaussian HMM)

The Gaussian HMM offers a number of advantages, most notably its ability to incorporate temporal features using delta coefficients [38]. This model can be seamlessly integrated with existing techniques and has shown significant improvement in continuous recognition tasks. It also provides a robust parametric representation of the data and excels in temporal modeling and segmentation [39]. However, the Gaussian HMM also faces challenges. The direct introduction of delta coefficients can be problematic, and there is potential for a resonance effect [38]. The performance of the model can be heavily dependent on the quality of the delta coefficients, and there is a noticeable lack of normalization. In addition, its effectiveness can be compromised if the size of the training data are not substantial enough [39].

#### 4.2.5. Naive Bayes

The Naive Bayes algorithm is revered for its computational efficiency and ability to quickly process large datasets [40]. It is unique in its incremental construction, which allows for easy updates and the inclusion of new cases [41]. Other advantages include the ability to reject uncertain classifications, the ability to modify utility functions, and the ability to compensate for class imbalances [42]. However, the independence assumption of Naive Bayes is its main limitation. Its static nature can sometimes lead to inaccuracies and, despite its efficiency, the model is limited by the size of the training set [40].

#### 4.2.6. Long Short-Term Memory (LSTM) Networks

The LSTM is a type of recurrent neural network that effectively overcomes the notorious gradient problems of traditional RNNs, allowing them to process long sequences without significant degradation [43]. This property, coupled with their design, gives them higher fitting and prediction accuracy for many tasks. However, they do have their own challenges. The training time for LSTMs can be significantly longer due to their complexity. In addition, they operate under certain naive assumptions that do not always match real-world scenarios [43].

#### 4.2.7. Artificial Neural Networks (ANNs)

ANNs, the precursors of the deep learning approach, are known for their profound capabilities. They have the intrinsic ability to recognise complex non-linear relationships between variables and can intuitively perceive interactions between predictor variables [44]. In addition, their design gives them fault tolerance and the ability to operate with incomplete knowledge [45]. Their parallel processing capability makes them highly scalable and efficient in certain applications. However, they are not without their challenges. The effectiveness of neural networks often depends on hardware specifications, which can make them hardware dependent. The behavior of a neural network can sometimes be opaque, leading to questions about interpretability. Determining the optimal network structure remains a difficult challenge, and the exact training time of the network can be unpredictable [45].

#### 4.2.8. Support Vector Classification (SVC)

In the case of SVC, a gentle introduction to Support Vector Machines (SVMs) seems desirable. SVMs are a set of related supervised learning methods [46], typically used for classification [47] and regression [48]. In addition, by offering a unique solution backed by a strong regularization function, SVMSs are particularly suited to classification problems that may be poorly conditioned [49]. A key strength lies in their ability to use a hyperplane with maximum margin to differentiate classes of data, ensuring commendable overall performance.

However, SVMs have inherent limitations. A notable concern is the computational cost they incur when deployed on large datasets. As the training kernel matrix grows quadratically with data size, training becomes progressively slower [50]. This scaling issue makes SVMs less suitable for classifying extensive datasets due to both time and memory constraints. Additionally, SVMs can exhibit subpar accuracy when confronted with imbalanced datasets [50].

Support Vector Classification (SVC) is an SVM algorithm for two-group classification problems [51]; it has the ability to effectively perform non-linear classification by exploiting the kernel trick of implicitly mapping inputs into high-dimensional feature spaces [49]. In addition, SVC is particularly praised for its ability to diagnose faults, adding another layer of utility to its application. However, it is not without its shortcomings. SVC classifiers can be computationally expensive and do not scale optimally [52]. Their training convergence can be slow when faced with large datasets, and they can require a significant number of support vectors, sometimes as many as half the size of the dataset. Especially in non-linear classification scenarios with large datasets, this property can hinder their effectiveness.

#### 4.2.9. Multi-Layer Perceptron (MLP)

The MLP is a basic neural network model known for its streamlined nature. With few parameters, it is suitable for those without extensive prior knowledge, and its algorithms are easy to implement [53]. One of its main advantages is its ability to construct the required decision function directly from a given data set during the learning process. This learning process is inherently adaptive, meaning that MLPs can autonomously learn solutions directly from the data being modeled. However, MLPs have their drawbacks. Effective learning often requires a significant number of patterns and iterations. Determining the optimal number of neurons and layers in their hidden layer can be challenging, often requiring numerous trials under varying conditions. Furthermore, the opaque nature of MLPs means that they do not elucidate the causality of events within the system, although some clarity can be derived through sensitivity analysis [53].

#### 4.2.10. Logistic Regression

Logistic regression is a staple of statistical modeling and machine learning. Its advantages lie in its inherent low variance, making predictions more consistent across different samples [54]. Another salient feature is its ability to provide probabilities for outcomes, offering more nuanced insights beyond binary predictions. It is relatively easy to use, and its training process is usually efficient and does not require extensive computational time. However, there are limitations to consider. While it is fundamentally designed for binary classification, adapting it to multi-class data requires specific modifications and techniques. In addition, its performance may be compromised when dealing with correlated attributes as it may not accurately capture the underlying patterns in such cases [54].

#### 4.2.11. Support Vector Regression (SVR)

The SVR is an adaptation of Support Vector Machines (SVM) tailored to regression problems by introducing an alternative loss function that allows one to effectively model continuous outcomes [49]. In small-sample scenarios, where the dimensionality of the data exceeds the number of samples, a careful application of machine learning theory (MLT) can often yield better results than other methods in determining the optimal hyperparameters of an SVM [55]. Theoretical methods have the distinct advantage over hold-out methods of using the entire dataset for both model training and generalization error estimation, which is particularly important when data availability is sparse. However, there are a few obstacles. The MLT-based approach can exhibit pessimistic behavior due to the Maximal Discrepancy method, and its computational complexity is not better than resampling-based techniques. Furthermore, reducing the size of the training set can drastically affect the reliability of the classifier [55].

#### 4.2.12. Recurrent Neural Networks (RNNs)

RNNs have carved out a niche in the field of deep learning, especially when it comes to handling sequential data. Their hallmark is their unique architecture, in which each cell retains memory of its predecessors, allowing the model to process data in time steps, a feat unattainable by many other machine learning models [56]. This inherent memory makes RNNs well suited to tasks where patterns recur over time, giving them an edge in recognizing time-dependent patterns [57]. However, they are not without their challenges. One prominent problem stems from their sequential nature, where continuous multiplication during forward propagation across time steps can lead to long-term dependencies during backpropagation. This can lead to the notorious “vanishing gradient” problem [58]. Furthermore, the need for associated hidden unit targets for each pattern limits their usefulness in online learning scenarios where patterns are typically encountered only once [59].

#### 4.2.13. 1D Convolutional Neural Networks (1D-CNNs)

The 1D CNNs are tailored versions of CNNs adapted to one-dimensional sequential data. Their strength lies in their ability to learn complex patterns through feature extraction, making them adept at processing sequential data [60]. They are also adept at handling high-dimensional inputs and often offer computational efficiency, especially when compared to more complex models. However, they have their own challenges. They are not well suited to managing variable-length inputs, which can limit their applicability in certain domains. In addition, LSTMs may be a better choice than 1D CNNs for tasks that require the maintenance of long-term dependencies or memory [60].

#### 4.2.14. The k-Nearest Neighbors (kNN)

The k-Nearest Neighbours (kNN) algorithm stands out in the world of machine learning for its simplicity and intuitive approach. It demonstrates robustness to noisy training data and often delivers effective results when the training dataset is extensive [61]. In addition, kNN shows commendable performance in scenarios where the training sample includes a plethora of class labels [62]. However, kNN is not without its limitations. Choosing a small value for *k* can make the algorithm overly sensitive to noise [63]. On the other hand, choosing a very large *k* can cause the computational cost to “skyrocket”. The algorithm’s efficiency also takes a hit when dealing with high-dimensional datasets, often resulting in significant slowdowns. Another significant drawback is its inability to efficiently accommodate online learning scenarios as each pattern to be learned requires associated targets for the hidden units, making the technique unsuitable for cases where patterns occur singularly [63].

#### 4.2.15. Decision Trees (DTs)

The DTs are graphical representations used for classification and regression tasks in machine learning. They have notable advantages, including the ability to support incremental learning, which allows the model to learn progressively with each new piece of data [64]. In addition, decision trees are memory efficient, requiring less memory than some other machine learning models. They also show a commendable ability to handle noisy data, demonstrating resilience in such scenarios. However, they come with their own set of challenges. One of the main concerns is their long training time, especially for large datasets. Another limitation is the potential for a more convoluted representation of certain concepts due to the replication problem [64]. In cases with small sample sizes, decision trees can be prone to overfitting, resulting in over-classification or a model that is too tailored to the training data [65]. Furthermore, because they are non-parametric, they make no assumptions about the distribution of the data set, which can be either an advantage or a limitation depending on the application.

#### 4.2.16. Adaptive Boosting (AdaBoost)

AdaBoost is a machine learning algorithm that focuses on boosting the performance of weak classifiers. It is renowned for its low generalization error, making it a reliable choice for various classification tasks [66]. Moreover, it is computationally efficient, meaning that it can swiftly process large datasets without excessive resource demands. Another favorable attribute of AdaBoost is its adaptability; it can be easily modified to meet specific requirements or integrated with other learning algorithms, underlining its flexibility. However, like any tool, AdaBoost has its limitations. It has a noted sensitivity to outliers, meaning that anomalous data points can adversely affect its performance. Training the model can introduce substantial noise, potentially compromising its efficiency. The algorithm also has a preference for larger samples, limiting its effectiveness in scenarios with limited data. Furthermore, the compositions it generates can sometimes become “unwieldy” or overly complex, especially when integrating multiple weak learners [66].

#### 4.2.17. eXtreme Gradient Boosting (XGBoost)

XGBoost is a machine learning algorithm designed to improve and optimize gradient boosting. One of its key strengths is the bucketing technique it applies to features. By assigning the same weight to all buckets and only increasing the weight of the required feature buckets in each iteration, XGBoost effectively filters out superfluous features, resulting in an increase in classifier speed [67]. Based on tree-boosting machine learning algorithms, XGBoost ensures a more harmonious balance between bias and variance, resulting in a more optimal “bias-variance” trade-off. In addition, XGBoost shows excellent performance, especially on large datasets, and manages to be fast in execution, making it favorable for real-world applications [68].

On the other hand, XGBoost is not without its challenges. The depth of the method can be complicated, making it a daunting task for beginners or those unfamiliar with gradient boosting [67]. The models produced by XGBoost tend to have low bias but high variance, which can sometimes compromise generalization to unseen data. Finally, a significant drawback is the amount of computation required during the tuning phase. As parameter tuning becomes essential to optimize model performance, it can consume over 99.9% of computational resources, underlining its resource-intensive nature [68].

#### 4.2.18. Random Forest (RF)

Random Forest (RF) is an ensemble learning method that constructs multiple decision trees during training and returns the mode of the classes (classification) or the mean prediction (regression) of the individual trees for unseen data. It has many advantages, particularly when dealing with complex data sets. For example, RF is resilient to problems of information overlap (multicollinearity) and over-parameterization, typically caused by excessive covariates. Its design inherently protects against overfitting, making it possible to fit models with a significant number of covariates [69].

In addition, it simultaneously accounts for spatial autocorrelation and correlation with spatial environmental factors, eliminating the need to deal with them separately. Notably, RF models do not require stationarity assumptions, nor do they require transformations, anisotropy parameters, or even variogram fitting. This gives RF flexibility as there is no need to specify a functional form or identify potential interactions [70].

However, the model is not without its challenges. To many, RF can appear as a ’mysterious black box’, obscuring whether anomalies in the output maps are due to input data artefacts or inherent model limitations [69]. Despite its ability to handle spatial data, RF tends to overlook the spatial locations of observations, neglecting spatial autocorrelation not captured by covariates. A pitfall of using RF in a spatial context is the inclusion of northing and easthing as covariates. This can inadvertently produce linear boundaries on maps that reflect the layout of the sampling plan rather than capturing true spatial patterns. Finally, the flexibility offered by RF comes with a trade-off. The lack of equations correlating variables with estimated risk can present challenges when trying to interpret the complex relationships within the data [70].

#### 4.2.19. CatBoost

CatBoost is a gradient boosting algorithm that focuses primarily on categorical data, providing an advantage over other algorithms that require the conversion or fitting of such data prior to processing. One of its key advantages is its ability to automatically handle categorical data using statistical methods, thus eliminating the pre-fitting of categorical data required by other methods [71]. CatBoost is also designed to reduce over-fitting by optimizing its many input parameters. Unlike some competitors, CatBoost does not deal with categorical features during the processing time but effectively manages them during the training phase. Impressively, CatBoost maintains strong performance even when the data size is relatively small [71].

On the downside, even with its advances aimed at curbing overfitting, CatBoost, as a tree-based model, is not entirely immune to this problem. Tree-based models inherently use a greedy algorithm that seeks optimal training accuracy. This can be a challenge when working with incomplete datasets. The algorithm may struggle to capture all of the non-linear relationships present, ultimately causing the model to overfit [72]. This highlights the importance of providing comprehensive data inputs to ensure the robustness and accuracy of the CatBoost model.

## 5. Performance Evaluation

This section is devoted to evaluating the performance of the methods in identifying anomalies. In the anomaly detection problem, we consider an anomaly to be an outlier, that is, an observation that does not appear to be consistent with other observations in the dataset [73]. In the context of our study, an anomaly is also understood as a signal that is too different from others, i.e., that is generated by a different mechanism or significantly affected by external factors.

Our empirical study is motivated by the core aspect that underlies the design and implementation of any anomaly detection method, which is effectiveness. In this sense, we pose the following research question: What is the most effective anomaly detection method for sensor data environments? As an example of this type of data, we have chosen the data generated by the vibration (acoustic) sensor mounted on a railway carriage.

### 5.1. Input

The dataset was collected by acoustic sensors measuring parameters of a railway vehicle, saved in the CSV (Comma-Separated Values) format. The input set contains 3657 signals (observations). Each observation represents a consecutive measurement collected by a sensor at a specific time, along with additional information.

A signal is described by nine different attributes, presented and briefly described in Table 2. Moreover, to better understand the nature of the data, Table 3 below shows an example of a selected observation from the input dataset.

In our analysis, we will focus on two of them: ‘values’ (#6) and ‘vibration_alert’ (#7). While the former is an attribute that contains a time series of vibration data collected by the acoustic sensors, the latter is an attribute that classifies the vibration data into three categories:

- 0: Checked, No Anomalies Detected: This indicates that the vibration data were checked, and no anomalies were detected.
- 1: Anomaly Detection: Anomalies were detected in the vibration data, raising an alert.
- 2: Impossible to Determine: This category is used when there are issues with the data, such as missing or incorrect information.

### 5.2. Data Analysis

**Figure 2:**
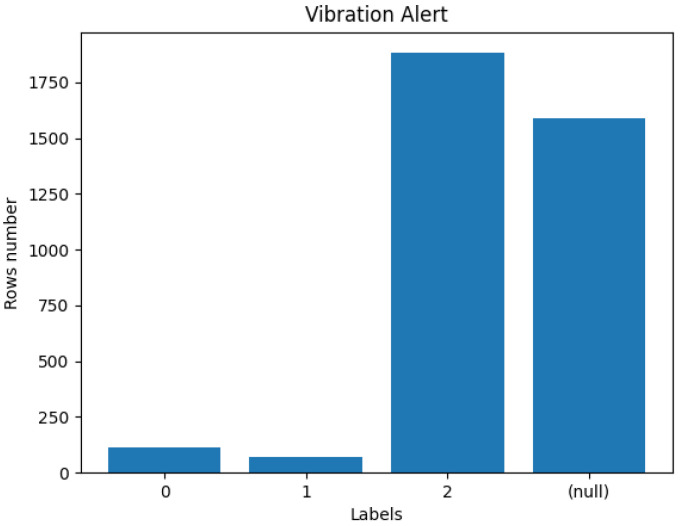
Vibration alert distribution.

There are 1882 observations in which the ‘vibration_alert’ attribute is assigned the value ‘2’. These instances indicate potential data quality concerns as they fall into the ‘Impossible to Determine’ category. Additionally, 1591 rows have the ‘vibration_alert’ attribute marked as ‘(null)’. This suggests that either the information for these instances is missing or not properly recorded. There are only 116 rows in which the ‘vibration_alert’ attribute is assigned the value ‘0’ and 68 rows in which the ‘vibration_alert’ attribute is assigned the value ‘1’. In summary, the distribution of the ‘vibration_alert’ attribute is depicted in Figure 2.

**Figure 3:**
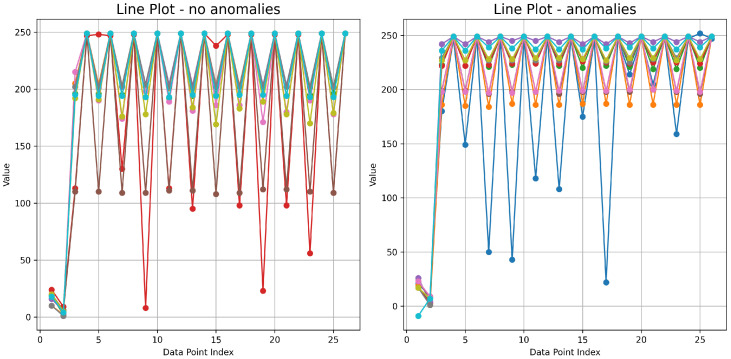
Time series data.

**Figure 4:**
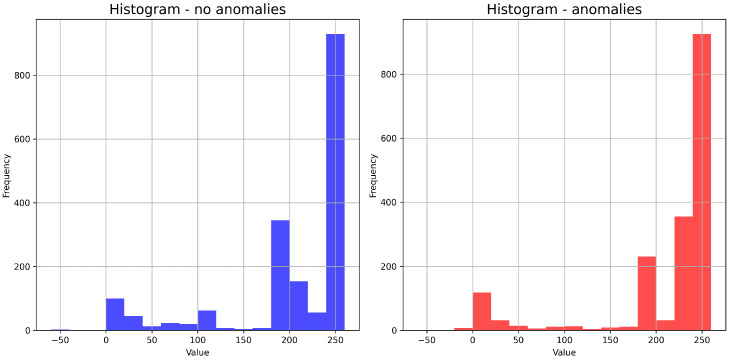
Values distribution—histogram.

Line plots Figure 3 have been generated by randomly selecting ten time series. At first glance, we can see that the scores are higher when there are anomalies. Histograms and box plots were also generated to check that the conclusion was correct. Since the number of series with recorded anomalies is relatively small (68), 68 samples were chosen for the histogram without anomalies. The minimum value is −60, and the maximum is 260. The number of bins is 16. On the basis of the histograms Figure 4, it can be seen that the vast majority of the values fall within the range [240, 260].

**Figure 5:**
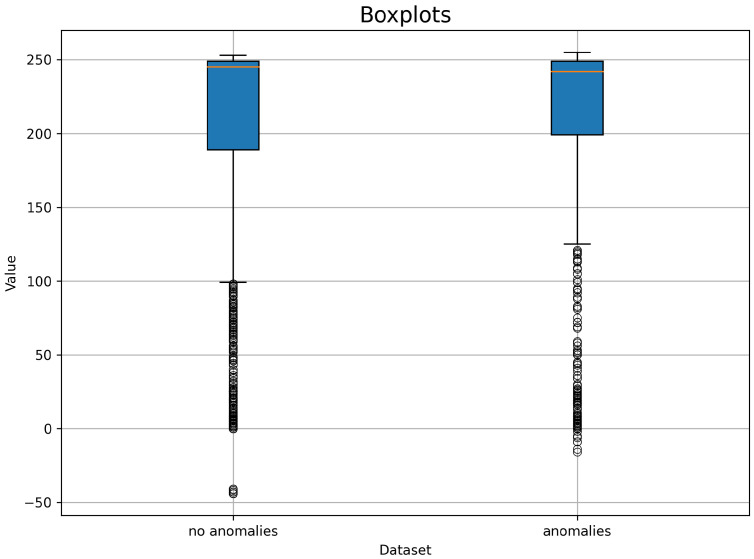
Values distribution—box plot.

However, it is worth noting that in the presence of an anomaly, a significant proportion of the values fall within the range [220, 240], whereas in the absence of an anomaly the values are distributed over a wider range of [200, 240]. The box plots in Figure 5 seem to confirm this judgement.

### 5.3. Data Preparation for Training Machine Learning Models

Before training the machine learning models, the data were carefully prepared. This data pre-processing phase involved several critical steps:

1. Data Cleaning. As part of the data cleaning process, rows containing ‘vibration_value’ equal to 2 or zero were removed from the dataset. This step was essential to maintain data integrity and ensure that the training data were free from inconsistencies or missing values that could compromise the quality of the models.
2. Normalization. This step aimed to subtract the mean (average) value of a feature from each data point and then divide it by the standard deviation. This method ensures that the values of different features are on a common scale, with a mean of 0 and a standard deviation of 1, making them suitable for different machine learning algorithms.
3. Additional Transformations. In addition to normalization, other transformations such as one-hot encoding were applied as needed. One-hot encoding was used to convert categorical variables into a binary matrix format, allowing machine learning models to work effectively with categorical data. This technique creates binary columns for each category within a categorical variable, ensuring that categorical information is appropriately represented in a numerical format.

After performing the above operations, the input data look as follows: from the time series (values), Table 4 presents a matrix that was created with dimensions of 184 × 26. From the labels (vibration_alert), a vector *y* was generated with dimensions of 184 × 1 (see Table 5 for details):

Having performed all of the necessary steps on a raw dataset with the aim of feeding machine learning models, below we shortly describe their implementations and settings.

### 5.4. Methods Implementation

In total, 19 methods were used to build the empirical studies. It should be noted that in order to maintain the replicability of the study, where possible, most of the machine learning models were built using a default configuration.

#### 5.4.1. Local Outlier Factor (LOF)

The LOF model is used to detect anomalies by assessing the local density of data points. It calculates the LOF score for each data point based on its density relative to its neighbours. The process starts by defining a neighbourhood around each data point, typically using the k-nearest neighbours algorithm. The LOF score for a point is then calculated by comparing the density of the point’s neighbourhood to the density of its neighbours. If a point has a significantly lower density than its neighbours, it is considered an outlier with a higher LOF score. In this way, LOF identifies anomalies by evaluating the local context of data points, making it effective for detecting outliers in complex datasets with varying densities.

The model was implemented using sklearn.neighbors.LocalOutlierFactor with the following parameters: n_neighbors = 20, algorithm = ‘auto’, leaf_size = 30, metric = ‘minkowski’, p = 2, metric_params = None, contamination = ‘auto’, novelty = True, and n_jobs = None.

#### 5.4.2. Isolation Forest (IF)

The IF is an anomaly detection algorithm that works by isolating anomalies within a data set. It does this by randomly selecting features and splitting values between the minimum and maximum values of those features, creating a binary tree structure through recursive splitting. The core idea of the algorithm is that anomalies that are rare and distant from the majority of data points tend to require fewer splits to isolate. During training, the isolation forest builds many such isolation trees. The final anomaly score for a data point is determined by averaging the path lengths across all trees, with shorter paths indicating a higher likelihood that the point is an anomaly.

The model was implemented using sklearn.ensemble.IsolationForest with the following parameters: n_estimators = 100, max_samples = ‘auto’, contamination = ‘auto’, max_features = 1.0, bootstrap = False, n_jobs = None, random_state = None, verbose = 0, and warm_start = False.

#### 5.4.3. Gaussian Hidden Markov Model (GHMM)

The Gaussian Hidden Markov Model (GHMM) is a statistical model used for anomaly detection. In this context, data are processed by GHMM by first representing it as a sequence of observations over time. Each observation is assumed to be generated from a Gaussian distribution. The GHMM consists of hidden states that capture the underlying dynamics of the data. These states are not directly observable but influence the generation of observations. The model learns transition probabilities between hidden states, reflecting how the data evolve over time. Anomalies are detected by evaluating the likelihood of a given sequence of observations under the GHMM. If a sequence has a significantly low likelihood according to the model, it suggests that the data follow a different pattern than what the GHMM has learned. This deviation from the learned behavior is indicative of an anomaly. GHMMs are particularly useful for time-series data where anomalies may manifest as deviations from the expected temporal patterns.

The model was implemented using hmmlearn.hmm.GaussianHMM with the following parameters: n_components = 2, covariance_type = ‘diag’, min_covar = 0.001, startprob_prior = 1.0, transmat_prior = 1.0, means_prior = 0, means_weight = 0, covars_prior = 0.01, covars_weight = 1, algorithm = ‘viterbi’, random_state = 42, n_iter = 50, tol = 0.01, verbose = False, params = ‘stmc’, init_params = ‘stmc’, and implementation = ‘log’.

#### 5.4.4. Naive Bayes

In the context of anomaly detection, the Naive Bayes model processes data by applying probabilistic reasoning. It assumes that the features in the data are conditionally independent given the class labels, which is why it is called “naive”. To use Naive Bayes for anomaly detection, the model must first be trained on a dataset containing both normal and anomalous instances. During training, it calculates the probability distributions of the features for each class (normal and anomalous). These distributions represent how the features in each class are expected to behave. When it comes to detecting anomalies in new data, Naive Bayes calculates the probability of observing a particular set of feature values, given both the normal and anomalous classes. It then uses Bayes’ theorem to calculate the posterior probability that an instance belongs to the anomaly class. If this posterior probability exceeds a predefined threshold, the instance is classified as an anomaly.

The model was implemented using sklearn.naive_bayes.GaussianNB with the following parameters: priors = None, var_smoothing = $1\times 10^{-9}$.

#### 5.4.5. Support Vector Classification (SVC)

Support Vector Classification (SVC) can be considered as an extension of the traditional Support Vector Machine (SVM), with a primary emphasis on binary classification problems. Like SVM, SVC leverages the fundamental principles of margin maximization and the use of support vectors, but its unique design and tuning make it an ideal choice for handling classification tasks. SVC excels in situations where the goal is to categorize data into two distinct classes, such as anomaly detection.

The model was implemented using sklearn.svm.SVC with the following parameters: C = 1.0, kernel = ‘rbf’, degree = 3, gamma = ‘scale’, coef0 = 0.0, shrinking = True, probability = False, tol = 0.001, cache_size = 200, class_weight = None, verbose = False, max_iter = −1, decision_function_shape = ‘ovr’, break_ties = False, and random_state = None.

#### 5.4.6. Support Vector Regression (SVR)

Support Vector Regression (SVR) finds a valuable application in anomaly detection when considering its capabilities beyond traditional regression tasks. While SVM is primarily associated with classification and SVR with regression, both can be adapted for anomaly detection purposes. In the context of anomaly detection, SVR departs from its traditional regression objectives. Instead of fitting a hyperplane that best captures the data distribution, the goal of SVR is to identify anomalies or outliers that do not fit the expected patterns within the data set. SVR uses the principle of margin maximization to determine the threshold for what constitutes an anomaly. Data points that exceed this threshold are considered anomalies as they represent a significant deviation from the established norm.

The model was implemented using sklearn.svm.SVR with the following parameters: kernel = ‘rbf’, degree = 3, gamma = ‘scale’, coef0 = 0.0, tol = 0.001, C = 1.0, epsilon = 0.1, shrinking = True, cache_size = 200, verbose = False, and max_iter = −1.

#### 5.4.7. One-Class SVM

One-Class SVM is designed for anomaly detection in situations where only one class (the normal class) is represented in the training data. It learns to model the distribution of normal data points and identifies anomalies as data points that deviate significantly from this learned distribution. It is particularly useful when dealing with unbalanced datasets or when there is a lack of labelled anomaly data.

The model was implemented using sklearn.svm.OneClassSVM with the following parameters: kernel = ‘rbf’, degree = 3, gamma = ‘scale’, coef0 = 0.0, tol = 0.001, nu = 0.5, shrinking = True, cache_size = 200, verbose = False, and max_iter = −1.

#### 5.4.8. Logistic Regression

Logistic regression is initially trained on a labelled dataset consisting of both normal and abnormal instances. During this training phase, the model learns to establish a decision boundary that effectively separates the two classes. The goal of the model is to calculate the probability that a given data point belongs to the positive class (anomaly) based on its features. To achieve this, Logistic Regression uses the logistic function (sigmoid) to transform a linear combination of the input features into a value between 0 and 1. This value represents the estimated probability that the instance is an anomaly. Once trained, the logistic regression model can be used for anomaly detection by applying it to new, unlabelled data. For each data point, the model calculates the probability, and if it exceeds a predefined threshold, the point is classified as an anomaly; otherwise, it is classified as normal.

The model was implemented using sklearn.linear_model.LogisticRegression with the following parameters: penalty = ‘l2’, dual = False, tol = 0.0001, C = 1.0, fit_intercept = True, intercept_scaling = 1, class_weight = None, random_state = None, solver = ‘lbfgs’, max_iter = 100, multi_class = ‘auto’, verbose = 0, warm_start = False, n_jobs = None, and l1_ratio = None.

#### 5.4.9. K-Nearest Neighbors (KNN)

The *k*-Nearest Neighbors (KNN) model for anomaly detection processes data by calculating the Manhattan distances between each data point in the test set and all data points in the training set. It selects the k nearest neighbors for each test data point based on these distances. The labels of these nearest neighbors from the training set are aggregated, and the test data point is classified based on the most frequent label among the neighbors. KNN determines anomalies by considering the consensus of labels among the nearest neighbors.

The KNN model is configured to use k = 2 nearest neighbors and the Manhattan distance metric for anomaly detection.

#### 5.4.10. Decision Tree

The decision tree is trained on a labelled dataset containing examples of both normal and abnormal cases. During training, the model learns to create a tree structure where each internal node represents a decision based on a feature and each leaf node represents a class label, which in this case would be normal or anomalous. The decision tree algorithm aims to construct a tree that effectively divides the data into subsets that are as pure as possible in terms of class labels. In other words, it seeks to minimize impurity or maximise information gain at each node when making decisions about which features to split on. To detect anomalies in new, unlabelled data, the model traverses the tree from the root node down to a leaf node, making decisions based on the feature values of the data point being evaluated. The last leaf node reached determines the classification of the data point, whether it is normal or an anomaly.

The model was implemented using sklearn.tree.DecisionTreeClassifier with the following parameters: criterion = ‘gini’, splitter = ‘best’, max_depth = None, min_samples_split = 2, min_samples_leaf = 1, min_weight_fraction_leaf = 0.0, max_features = None, random_state = None, max_leaf_nodes = None, min_impurity_decrease = 0.0, class_weight = None, and ccp_alpha = 0.0.

#### 5.4.11. Random Forest

In the context of anomaly detection, the Random Forest algorithm processes data through an ensemble of decision trees to identify anomalies within a dataset. Random Forest is initially trained on a labelled dataset containing examples of both normal and anomalous cases. During training, the model creates an ensemble of decision trees, each trained on a subset of the data and a subset of the features. This ensemble approach helps reduce overfitting and improves generalization. Each decision tree within the random forest independently makes predictions based on the input data. When it comes to anomaly detection, the individual decision trees classify data points as either normal or anomalous based on their internal learned rules. To make a final anomaly prediction for a given data point, the Random Forest combines the predictions of all the decision trees in the ensemble. This is done by majority voting, where the class predicted by the majority of trees becomes the final prediction. Anomalies are often detected when a significant proportion of the decision trees in the Random Forest ensemble classify a data point as an anomaly. The idea is that anomalies are less likely to fit the common patterns captured by most of the decision trees, resulting in a consensus among the trees for classifying an anomaly.

The model was implemented using sklearn.ensemble.RandomForestClassifier with the following parameters: n_estimators = 100, criterion = ‘gini’, max_depth = None, min_samples_split = 2, min_samples_leaf = 1, min_weight_fraction_leaf = 0.0, max_features = ‘sqrt’, max_leaf_nodes = None, min_impurity_decrease = 0.0, bootstrap = True, oob_score = False, n_jobs = None, random_state = None, verbose = 0, warm_start = False, class_weight = None, ccp_alpha = 0.0, and max_samples = None.

#### 5.4.12. AdaBoost

In the context of anomaly detection, AdaBoost combines multiple weak classifiers trained on labelled data. It assigns weights to data points, giving more weight to misclassified data points. The algorithm trains weak classifiers iteratively, adjusting weights and focusing on difficult instances. Anomalies are identified when data points with high weights are difficult to classify.

The model was implemented using sklearn.ensemble.AdaBoostClassifier with the following parameters: estimator = None, n_estimators = 50, learning_rate = 1.0, algorithm = ‘SAMME.R’, and random_state = None.

#### 5.4.13. XGBoost

XGBoost processes data using an ensemble of decision trees. it is first trained on a labelled dataset containing normal and anomalous instances. During training, XGBoost creates a collection of decision trees, often flat to avoid overfitting. Each decision tree independently predicts whether a data point is normal or an anomaly based on its learned rules. To make a final prediction, XGBoost combines the outputs of all the decision trees, often by averaging their predictions. Anomalies are identified when a significant proportion of the decision trees in the XGBoost ensemble classify a data point as an anomaly.

The model was implemented using xgboost.XGBClassifier with the default parameters.

#### 5.4.14. CatBoost

In the area of anomaly detection, CatBoost processes data using a gradient boosting algorithm that focuses on improving the performance of decision trees. It begins with supervised training on a labelled dataset containing both normal and anomalous instances. During training, CatBoost constructs an ensemble of decision trees that adapts to the complexity of the data. Each decision tree in the CatBoost ensemble independently evaluates data points, classifying them as normal or anomalous based on learned patterns. These individual tree outputs are then combined to produce a final prediction. Anomalies are identified when a significant proportion of the decision trees within the CatBoost ensemble classifies a data point as an anomaly. The collective agreement between the trees highlights data instances that deviate from expected patterns.

The model was implemented using catboost.CatBoostClassifier with the default parameters.

#### 5.4.15. Artificial Neural Network (ANN)

Artificial Neural Networks (ANNs) are a class of deep learning models inspired by the structure and function of the human brain. In the context of anomaly detection, ANNs are versatile and can be applied to different types of data, including tabular and structured data. They consist of interconnected layers of artificial neurons, with each layer performing specific computations on the input data. ANNs are able to learn complex patterns and relationships within data, making them effective for anomaly detection tasks, especially when the underlying patterns may not be explicitly temporal.

The model starts with an input layer of 8000 neurons using the ReLU activation function. The subsequent layers consist of three hidden layers, each with two neurons and ReLU activation. The final layer of the model is a single neuron with sigmoid activation. The model is trained using stochastic gradient descent ‘sgd’ as the optimizer and ‘binary_crossentropy’ as the loss function.

#### 5.4.16. Multilayer Perceptrons (MLPs)

Multilayer Perceptrons, commonly known as MLPs, are a fundamental class of neural networks widely used for anomaly detection. They use a feedforward architecture with input, hidden, and output layers, with multiple neurons in each layer. This architectural flexibility allows MLPs to capture complex and non-linear relationships between features, making them adaptable to high-dimensional data. They excel at detecting anomalies that do not have a specific temporal sequence but are instead anomalies scattered across different dimensions or features.

The model was implemented using sklearn.neural_network.MLPClassifier with the following parameters: hidden_layer_sizes = (100), activation = ‘relu’, solver = ‘adam’, alpha = 0.0001, batch_size = ‘auto’, learning_rate = ‘constant’, learning_rate_init = 0.001, power_t = 0.5, max_iter = 200, shuffle = True, random_state = None, tol = 0.0001, verbose = False, warm_start = False, momentum = 0.9, nesterovs_momentum = True, early_stopping = True, validation_fraction = 0.1, beta_1 = 0.9, beta_2 = 0.999, epsilon = $1\times 10^{-8}$, n_iter_no_change = 10, and max_fun = 15,000.

#### 5.4.17. Recurrent Neural Networks (RNNs)

Recurrent Neural Networks (RNNs) specialize in processing sequential data, making them valuable for anomaly detection tasks involving temporal patterns. Unlike MLPs, RNNs incorporate recurrent connections, allowing them to maintain a hidden state that preserves information from previous time steps. This property enables RNNs to capture dependencies over time. However, RNNs may face challenges in capturing long-range dependencies as effectively as more advanced models such as LSTMs.

This neural network model is designed for binary classification tasks, featuring a SimpleRNN layer with 50 ReLU-activated neurons, Dense output layer, ‘adam’ optimization, and ‘binary_crossentropy’ loss.

#### 5.4.18. Long Short-Term Memory (LSTM)

Long Short-Term Memory (LSTM) networks are a variant of RNNs specifically designed to address the vanishing gradient problem and capture long-term dependencies in sequential data. LSTMs use memory cells that can store and update information over time, making them exceptionally well suited to modeling complex temporal relationships. This specialization makes LSTMs a powerful choice for detecting anomalies in sequential data with complex temporal dependencies.

The model consists of two LSTM layers of 64 units each, and dropout layers with a dropout rate of 0.2 are inserted after each LSTM layer to prevent overfitting. A RepeatVector layer is included to repeat the LSTM output sequence 26 times to match the input sequence length. The model ends with a TimeDistributed Dense layer. The model uses stochastic gradient descent (‘sgd’) as the optimizer and ‘binary_crossentropy’ as the loss function.

#### 5.4.19. One-Dimensional Convolutional Neural Networks (1D-CNNs)

One-Dimensional Convolutional Neural Networks (1D-CNNs) are tailor-made for processing one-dimensional sequences, such as time series or sequential sensor data. They use convolutional layers to learn local patterns and features within the data. While lacking the explicit temporal modeling capabilities of RNNs and LSTMs, 1D CNNs excel at capturing local features and are particularly effective at detecting anomalies in sequential data characterized by short-term dependencies and local irregularities.

The model has three Conv1D layers. The first layer has 64 filters, a kernel size of 3, and uses the ReLU activation function. The subsequent Conv1D layers have 128 and 256 filters, also using ReLU activation. After each Conv1D layer, a MaxPooling1D layer with a pool size of 2 is applied to downsample the data. After the last MaxPooling1D layer, a Flatten layer is included to transform the output into a one-dimensional vector. The model contains two Dense layers. The first Dense layer consists of 128 neurons with ReLU activation, while the second Dense layer has a single neuron with sigmoid activation. The model uses the ‘adam’ optimizer and the ‘binary_crossentropy’ loss function.

### 5.5. K-Fold Cross-Validation

In our case, K-fold cross-validation, presented in Table 6, was used as a technique to increase the reliability and robustness of our experimental results. The data set was divided into five equally sized subsamples. Each of these subsamples, or “folds”, played a different role in the cross-validation process, and the technique worked as follows:

1. Data Splitting. The dataset was first divided into five parts of approximately equal size, with each part acting as a fold. This division ensured that the distribution of data across the folds was maintained as far as possible.
2. Model Training and Testing. The cross-validation process was iterated five times, with each iteration using a different fold as the test set, while the remaining four folds collectively served as the training set. Each fold had the opportunity to be the test set once, while the model was trained on the rest of the data.
3. Performance Evaluation. After training on one set and testing on another, the model’s performance metrics were recorded for that particular iteration. This step ensured that the model’s performance was assessed comprehensively across different parts of the dataset.
4. Average Performance. To provide a more robust and reliable estimate of the model’s performance, the performance metrics from all five iterations were averaged. This average provided a single, representative measure of the model’s effectiveness at predicting outcomes.

In summary. After the five-fold cross-validation, with the aim of preserving the generalizability and stability of the models, the metric evaluation can be carried out.

### 5.6. Results

#### 5.6.1. Evaluation Metrics

In order to determine the most effective method, it is essential to establish a metric for comparing the different models. Accuracy (1) was chosen as the primary metric for evaluating the effectiveness of each model. Although this metric is intuitive and straightforward in analysis and interpretation [74], to avoid being misleading, Precision (2), Recall (3), F1 Score (4), and False Positive Rate (FPR) (5) are also included to provide a more nuanced assessment of model performance. These metrics capture the trade-offs between correctly identifying anomalies (true positives, TP), avoiding the misclassification of normal behavior as anomalous (false positives, FP), and not missing actual anomalies (false negatives, FN). The equations of the applied metrics are given below.

(1) $$\mathrm{Accuracy}=\frac{\mathrm{Number}\;\mathrm{of}\;\mathrm{correctly}\;\mathrm{classified}\;\mathrm{samples}}{\mathrm{Total}\;\mathrm{number}\;\mathrm{of}\;\mathrm{samples}\;\mathrm{in}\;\mathrm{the}\;\mathrm{test}\;\mathrm{set}}\times 100\%$$

(2) $$\mathrm{Precision}=\frac{\mathrm{Number}\;\mathrm{of}\;\mathrm{correct}\;\mathrm{positive}\;\mathrm{predictions}}{\mathrm{Total}\;\mathrm{number}\;\mathrm{of}\;\mathrm{positive}\;\mathrm{predictions}}\times 100\%$$

(3) $$\mathrm{Recall}=\frac{\mathrm{Number}\;\mathrm{of}\;\mathrm{correct}\;\mathrm{positive}\;\mathrm{predictions}}{\mathrm{Number}\;\mathrm{of}\;\mathrm{actual}\;\mathrm{positive}\;\mathrm{samples}}\times 100\%$$

(4) $$F1\;\mathrm{Score}=2\times \frac{\mathrm{Precision}\times \mathrm{Recall}}{\mathrm{Precision}+\mathrm{Recall}}\times 100\%$$

(5) $$\mathrm{False}\;\mathrm{Positive}\;\mathrm{Rate}\;\left(\mathrm{FPR}\right)=\frac{\mathrm{Number}\;\mathrm{of}\;\mathrm{incorrect}\;\mathrm{positive}\;\mathrm{predictions}}{\mathrm{Total}\;\mathrm{number}\;\mathrm{of}\;\mathrm{actual}\;\mathrm{negative}\;\mathrm{samples}}\times 100\%$$

#### 5.6.2. Method Comparison

**Figure 6:**
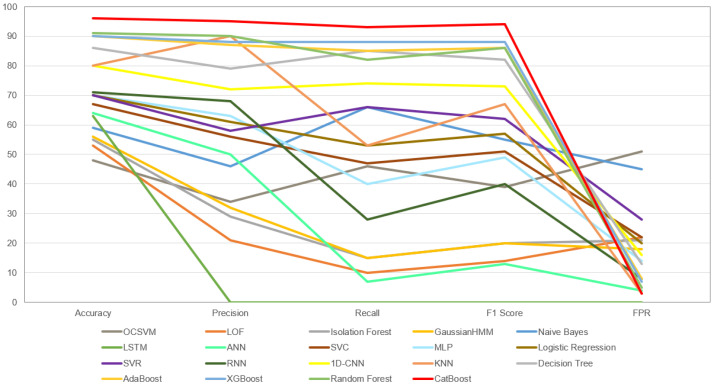
Plots of the model’s evaluation metrics.

Table 7 and Figure 6 presents all the implemented models and the performance of each of them. Based on the provided data on anomaly detection models and the comprehensive set of metrics, we can draw several conclusions:

1. The most effective model in terms of applied metrics is CatBoost, with an impressive accuracy of 96% and an F1 Score of 94%. This suggests that CatBoost is particularly well-suited for detecting anomalies in the given dataset.
2. Among the traditional machine learning models, Decision Tree, AdaBoost, XGBoost and Random Forest have relatively high accuracy percentages, ranging from 86% to 91%. These models also maintain commendable Precision and Recall, suggesting their effectiveness in balancing the identification of anomalies with minimizing false alarms.
3. Models such as OCSVM, LOF, Isolation Forest, and GHMM, with lower accuracy scores of 48% and 56%, also exhibit significant weaknesses across other metrics, highlighting their limited utility for this specific anomaly detection task.
4. Deep learning models show varied performance, with recursion-based and simple ANN models underperforming across all metrics, while 1D-CNN achieves the highest F1 score of 73% within this group, indicating its capability to balance Precision and Recall effectively.

In summary, the most effective model for anomaly detection on this dataset is CatBoost, closely followed by ensemble methods, viz: AdaBoost, XGBoost, and Random Forest. The least effective models are OCSVM and LOF. The deep learning models, particularly the more complex ones, did not perform as well due to the small size of the dataset, which confirms the importance of dataset size in training deep learning models. In contrast, 1D-CNN showed relatively better performance, potentially due to its simpler architecture. These findings underscore the need to match the model complexity with the available data volume to ensure effective anomaly detection.

## 6. Discussion

This section discusses the study’s contributions and limitations, as well as its theoretical and practical implications, to expound on the results obtained, analyze the potential ramifications of the conclusions, and explore future research opportunities in the field.

### 6.1. Study Contributions

In the first part of our study, we applied a well-recognised and valued systematic review methodology to enable an in-depth analysis of the literature in a process that is inherently reproducible and transparent. In this sense, as a first contribution, our paper presents the state of the art methods dedicated to anomaly detection in sensor data environments (Section 4.1). In addition, we also present a classification of these methods into taxonomic groups according to their identified similarities (Table 1). The second contribution is an in-depth analysis of the most commonly used methods in this field, highlighting their advantages and disadvantages (Section 4.2). In our opinion, such knowledge is valuable for data analysts, who deal with data sets of varying complexity and structure, looking for anomalous, irregular, false, or significantly affected observations obtained from sensor devices. In a broader sense, due to their generic nature, the identified methods can be applied to other diverse areas ranging from finance and banking (fraud detection, risk management) to healthcare (disease outbreak detection, patient monitoring), industrial production and manufacturing (equipment and machinery monitoring, quality control), research (data cleaning, experimental result validation), and utilities and infrastructure (energy consumption, water treatment), to name but a few. It would therefore be wise to assert that such a contribution is particularly useful when claiming its value.

In the second part of our study, we conducted a series of controlled experiments on a dataset to determine the effectiveness of the anomaly detection methods. This research, which is empirical in nature, contributes to the body of knowledge by providing empirical evidence on the performance of the methods in detecting anomalies in the sensor data environment. Specifically, the results show that ensemble methods, including CatBoost, Random Forest, XGBoost, and AdaBoost, performed better than others, with CatBoost showing the highest level of accuracy and F1 Score (Table 7). There are many reasons for this, including the fact that ensembles are less sensitive to the weaknesses of a single model by combining and aggregating the results of multiple models. In addition, ensembles can generalize better to unseen data, especially if the individual models have been trained on different subsets or under different conditions. Importantly, if individual models have certain biases, aggregating their predictions can offset these biases to some extent, leading to more balanced and accurate anomaly detection.

### 6.2. Study Limitations

It is clear that the process of study selection depends on the search strategy adopted, the literature sources selected, the inclusion and exclusion criteria, the quality of criteria, and that certain limitations are inevitable. Similar to other systematic reviews, a number of threats to the validity and legitimacy of the current study were identified. These threats were mainly in two aspects. These limitations relate to the selection of data sources and possible bias in the extraction and analysis of data.

Firstly, although it is a common scientific guideline to use several databases to search for relevant papers, we have chosen to use only one database, Scopus. It should be noted that Scopus covers more than 25,100 titles (including over 23,452 peer-reviewed journals) from approximately 5000 publishers [75] and thus offers a wider range of academic disciplines than Web of Science [76]. Moreover, Scopus is claimed to be the most authentic, comprehensive [77], and complete database of global scientific research [78], with 1.4 billion citations and 16 million author records [79]. In consequence, with some level of confidence, one can assume that the representative population of papers underwent investigation, where only few were not available.

Secondly, it is always necessary to provide a clear but concise certification of the search strategy used to facilitate the replication of the search at a later stage of the study. In our study, as a first step, we included a filter of titles, keywords, and abstracts of publications and used a predefined search string extracted from the research questions. However, to make the study feasible, we also used an additional keyword derived from the topic of the study. These keywords were chosen arbitrarily as the terms for each research question. To this end, we also limited our choice of publications to those written in English, excluding by default relevant studies that were not written in English, as our language skills were limited to one foreign language. Today, this restriction is a common practice and considered acceptable and non-invasive [80].

In addition, undeniably, more results could have been found if the search net had been cast wider. By excluding gray literature such as theses and dissertations, technical reports, working notes, white papers, and workshop and symposium papers [81], we also denied ourselves the opportunity to identify other relevant methods that may have been overlooked. On the other hand, gray literature is considered to be of low quality and is typically inaccessible [82].

Further considering the data analysis process, each discrepancy between individual researchers was the subject of intervention of others and, if necessary, discussed in an online meeting with all interested parties. In order to reach consensus during data extraction, the rapid resolution of disagreements between the data reviewer and the extractor was considered a priority. The researchers weighed all the issues in dispute and chose the best course of action at each point. In a broader sense, we adopted a researcher triangulation approach [83] to reduce individual bias. This allowed us to increase the validity and reliability of the study by providing a more complex and nuanced understanding of the possible interpretations of the research objects [84].

Turning now to the second part of our study, it is also important to acknowledge the limitations of the experimental design and implementation settings. By its very nature, validity in experimental research ensures that the results of an experiment accurately reflect the real-world situation they are intended to represent. Threats to validity can compromise the integrity and generalizability of research findings. Broadly speaking, threats to validity can be categorized into four main types: internal validity, external validity, construct validity, and statistical inferential (conclusion) validity [85]. These four types are discussed below.

Internal validity is defined as the extent to which the observed results represent the truth in the population we are studying and thus are not due to methodological errors [86]. Here, two separate aspects should be distinguished and considered, namely, the input dataset, and the implementations and settings of the anomaly detection methods used in the experiments. Firstly, an external company collected and processed a dataset in a controlled experiment using a physical sensor attached to a railway wagon. In addition, the metadata file was also shared, providing the necessary means to understand certain attributes and assess their relevance. We had no doubts about the reliability and validity of the data, given the reputation of the data provider. Nevertheless, the view of the data can be described in terms of a “black box” approach, without any knowledge of the internal settings and measures applied. The detailed information on the data collection process leaves a margin of dissatisfaction. However, it is not always possible to fully inform the reader due to the protection of intellectual properties.

Second, we used a well-recognised and extensively documented software package that has been developed, systematically updated, and verified by a multinational community of software developers and researchers. We are therefore confident that the results are trustworthy and that the conclusions drawn are ultimately correct. It should be emphasized that if a follow-up experiment uses a similar configuration, but on a later version of a package, and this is not clearly documented, it may be difficult for other researchers to reproduce the study.

In the context of the current study, external validity refers to the generalizability of the anomaly detection methods used to other data sets, settings or variables. Undeniably, the validity of inferences about the causal relationship identified is maintained over variations in both settings and measures used. However, the sample is not representative of the target population through randomization, and therefore the findings are only relevant to the sample of the study. Nevertheless, due to the uniformity of the physical components of railway wagons and the governance of the same natural laws, the research findings can be generalized to a reasonable extent to and across different environments and settings. Since external validity should be a goal pursued from the initial conception and design [87], other researchers who wish to replicate our study should adopt and adapt the most similar methods and settings to the current ones [88].

By definition, construct validity is the degree to which a test measures a theoretical construct that it is intended to measure [89]. In our opinion, the accuracy measure adequately measures the detection capability of the methods tested. On the other hand, since the source code of the methods used is publicly available, it can be assumed that their implementations are free of errors. Nevertheless, addressing construct validity is crucial for ensuring the meaningfulness and precision of the research findings [90]. It is an ongoing process that requires thoughtful development of existing and new measurement tools, pilot testing, and repeated validation in different settings and populations. Definitely, the development of open-source software is managed to ensure full transparency [91], high quality standards [92], and active collaboration among global community members [93], following the latest advances in research [94].

Last but not least, conclusion validity, which refers to how confident we can be that the treatment we used in a trial is related to the actual outcome we observed, does not affect our approach [95]. It should also be noted that we did not perform exhaustive hyperparameter optimization for all the methods used. This limitation may have resulted in suboptimal performance for some of the methods, potentially underrepresenting their true capabilities.

All of the above issues raised with respect to validity are recapitulated and briefly discussed below.

### 6.3. Future Research

To address the limitations identified in this study and to further our understanding of anomaly detection in vibration sensor data, several avenues for future research are worth exploring:

- Include more attributes. Extending the analysis to include more attributes could provide a more comprehensive understanding of the factors influencing anomaly detection.
- Hyperparameter optimization. Perform a thorough hyperparameter optimization for all methods to ensure that each is operating at peak performance.
- Test other datasets. Evaluating methods based on the other datasets will give a better understanding of their performance.
- Implement other methods. Investigate additional anomaly detection methods not considered in this study to assess their potential effectiveness in this particular domain.

While selecting appropriate settings for anomaly detection classifiers for the experimental setup is not an easy task, future research is expected to contribute in this area as well. In addition, there has recently been a growing interest in the development of models based on the eXplainable Artificial Intelligence (XAI) paradigm. Such an interpretable and explainable model can provide the necessary means to enable human users to understand the details behind the reasoning capacity of machine learning methods [96].

### 6.4. Unexpected Results

Despite the overall success of ensemble methods, we found a surprising and noteworthy result in the performance of unsupervised methods, including One-Class SVM (OCSVM), Local Outlier Factor (LOF), and Isolation Forest. These methods performed remarkably poorly in detecting anomalies, with OCSVM being the worst performer of all. This unexpected result is significant as it challenges conventional assumptions about the suitability of unsupervised methods for anomaly detection in vibration sensor data. A plausible interpretation of this unexpected result could be that unsupervised methods may struggle to capture the complex patterns and nuances present in vibration sensor data, which may require more sophisticated modeling techniques such as those provided by ensemble methods. Furthermore, the choice of hyperparameters may have played a critical role in the poor performance of the unsupervised methods. Optimization of the hyperparameters could potentially have improved their effectiveness.

## 7. Conclusions

In this paper, we have discussed various ways in which the problem of anomaly detection in railway sensor data environments has been solved and have attempted to evaluate the effectiveness of the most commonly used methods to date. The study is thus in line with the current trend towards increasing rail safety and, in a broader sense, improving the quality of rail services.

The study consisted of two parts. The first part was a systematic literature review with two research objectives: first, to identify time series anomaly detection methods applied to sensor device data, and second, to identify the advantages and disadvantages of these methods. A total of 71 methods were identified. In addition, we developed a classification by distinguishing five different types, namely, statistical (14), clustering (10), classification (29), based on Information Theory (2), and hybrid/other (16). From this result, 19 methods were recognized as the most popular and served as input for the second part of the study.

The second part was a controlled experiment to determine the effectiveness of the chosen methods. We used the scikit-learn machine learning library for the implementation as it is a powerful library with a huge number of possibilities. To assess the predictive performance of each method, a *k*-fold cross-validation approach (*k* = 5) was used to achieve the highest accuracy and consistency for each method. The results show that ensemble methods, in particular CatBoost, showed the highest accuracy (96%), while unsupervised methods, in particular OCSVM (48%), unexpectedly performed the worst.

CatBoost’s superior accuracy in detecting anomalies in the railway vibration dataset is due to its inherent strengths and the specific characteristics of the data. As a gradient boosting algorithm, CatBoost iteratively builds a series of decision trees, each refining the errors of its predecessor, enabling it to capture intricate patterns in complex datasets. This dataset, consisting of time-series vibration data and nominal indicators, benefits from CatBoost’s ability to seamlessly handle both categorical and numerical features without pre-processing. In addition, because railway vibration data can be inherently noisy, CatBoost’s built-in overfitting prevention techniques ensure that it does not misinterpret random fluctuations as significant patterns. In contrast, other models might either overfit to this noise or require extensive feature engineering to be effective. In the context of railway operations, where anomalies can signal critical safety issues, the robustness and Precision offered by CatBoost is invaluable.

Despite the limitations of our study, such as feature selection and hyperparameter tuning, these results contribute to the field of anomaly detection and can guide practitioners in selecting appropriate methods for monitoring and maintenance of railway wagons. Furthermore, the unexpected results regarding unsupervised methods highlight the need for further investigation and emphasize the importance of selecting the relevant techniques for specific data domains. Future research efforts should aim to address these limitations and explore the potential of other methods to improve the reliability and accuracy of anomaly detection in vibration sensor data.

Our research contributes to the advancement of railway safety by both identifying the advantages and disadvantages of existing anomaly detection methods and evaluating and comparing their performance on the real-world dataset. We believe that the adoption of these methods can lead to more reliable and responsive railway systems, in line with the industry’s commitment to a zero-defect paradigm, thus improving the safety and quality of rail transport on a global scale.

As more and more industrial sectors move towards a zero-defect paradigm [97,98,99], such research efforts seem to provide the necessary knowledge to also make this aspiration a reality for rail transport. In this sense, our work provides valuable insights into the methods of anomaly detection, ranging from statistical models to the most advanced machine learning algorithms, contributing to the ongoing research in this area. 

## Figures and Tables

**Table 1:** Identified anomaly detection methods, techniques, and systems.

Type	Methods	
Statistical (14)	Autoregressive Integrated Moving Average (ARIMA), Clock Drift, Covariance Matrix, Cumulative Sum, Exponential Weighted Moving Average (EWMA), Holt–Winters, Mahalanobis Distance, Markov Chains, Matrix Profile, Principal Component Analysis (PCA), Receiver Operating Characteristic Analysis, Robust Covariance, Singular Value Decomposition (SVD), and Wavelets Functions	
Clustering (10)	Density-Based Clustering Algorithm (DBSCAN), Graph-Based Approaches, Hierarchical Affinity Propagation, K-Harmonic Means (KHM), *k*–Means, *k*–Medoids, *k*–NN clusters, Ordering Points to Identify the Clustering Structure (OPTICS), Self-Organizing Maps (SOM), and Subspace Clustering	
Classification (29)	SVM (4)	SVC, SVR, One-Class SVM, SVDD
	Neural Networks (7)	Autoencoders, Bidirectional Recurrent Neural Network (BRNN), Convolutional Neural Network (CNN), Deep Neural Networks, Generative Adversarial Network (GAN), Long Short-Term Memory (LSTM), and Recurrent Neural Network (RNN)
	Ensemble Learning (8)	AdaBoost, CatBoost, DTBagg, dBoost, Gradient Boosting, LightGBM, Random Forests, and XGBoost
	Others (10)	Decision Trees, GaussianHMM, Isolation Forest, Levenberg–Marquardt Algorithm, Linear Discriminant Analysis, Local Outlier Factor, Logistic Regression, Naive Bayes, Quadratic Discriminant Analysis, and Synthetic Minority Oversampling Technique
Information Theory (2)	Entropy, Kullback—Leibler Divergence	
Hybrid/Others (16)	Artificial Immune Systems (AIS), Autoencoder and Incremental Clustering-Enabled Anomaly Detection, Cloud-Edge Indicator of Farming Anomalies (CEIFA), Deep Transfer Learning-Based Dual Temporal Domain Adaptation, Differential Evolution, Evolutionary Computation, Flocking Algorithm (FA), Fuzzy Combination of Outlier Detection techniques (FUCOD), Genetic Algorithms (GA), Hybrid Graph Transformer Network, Incremental Learning, Local Adaptive Multivariate Smoothing (LAMS), Mixed Deep-Learning-Based Methods Particle Swarm Optimization (PSO), Rough Sets, and Social Spider Optimization (SSO)	

**Table 2:** Dataset description.

No	Attribute	Data Type	Description
1	time	numeric	The timestamp indicating the exact time when the sensor readings were recorded
2	company_id	numeric	Identifier representing the company
3	sensor_sub_id	numeric	An identifier for the individual acoustic (vibration) sensor unit within the wheel system
4	battery	numeric	Information about the battery status of the sensor unit
5	status	nominal	Status indicator indicating the validity of the data
6	values	numeric	A time series of vibration data collected by the acoustic sensors
7	temperature_alert	nominal	Indicator for temperature-related alerts
8	vibration_alert	nominal	Indicator for vibration-related alerts
9	sensor_count	numeric	The number of sensor units

**Table 3:** The detailed values of a selected observation.

Attribute	Example
time	2080-01-07 13:08:12
company_id	55
sensor_sub_id	14
battery	3177
status	valid
values	[−1, 0, 2, 252, 239, 247, 219, 247, 220, 247, 221, 247, 220, 247, 220, 247, 219, 247, 218, 247, 220, 247, 219, 247, 219, 247]
temperature_alert	0
vibration_alert	1
sensor_count	1

**Table 4:** Time series data (values).

Index\Value	1	2	3	…	26
1	0.388767	−0.945435	0.308469	…	0.382076
2	0.516504	1.197810	0.157382	…	0.382076
3	0.197160	0.126187	0.526706	…	0.382076
…	…	…	…	…	…
184	0.644242	−1.659850	−2.260009	…	−1.885730

**Table 5:** Labels (vibration_alert).

Index	Vibration Alert
1	0
2	1
3	1
…	…
184	0

**Table 6:** K-fold Cross-validation schema.

	Fold 1	Fold 2	Fold 3	Fold 4	Fold 5
Iteration 1	Test	Train	Train	Train	Train
Iteration 2	Train	Test	Train	Train	Train
Iteration 3	Train	Train	Test	Train	Train
Iteration 4	Train	Train	Train	Test	Train
Iteration 5	Train	Train	Train	Train	Test

**Table 7:** Calculated accuracy of anomaly detection models.

Model	Accuracy (%)	Precision (%)	Recall (%)	F1 Score	FPR (%)
OCSVM	48	34	46	39	51
LOF	53	21	10	14	22
Isolation Forest	55	29	15	20	21
GaussianHMM	56	32	15	20	18
Naive Bayes	59	46	66	55	45
LSTM	63	0	0	0	0
ANN	64	50	7	13	4
SVC	67	56	47	51	22
MLP	70	63	40	49	14
Logistic Regression	70	61	53	57	20
SVR	70	58	66	62	28
RNN	71	68	28	40	8
1D-CNN	80	72	74	73	16
KNN	80	90	53	67	3
Decision Tree	86	79	85	82	13
AdaBoost	90	87	85	86	8
XGBoost	90	88	88	88	7
Random Forest	91	90	82	86	5
CatBoost	96	95	93	94	3

## Data Availability

Data available on request due to privacy restrictions.
